# Evaluation of a biopsychosocial education resource for mild traumatic brain injury: a mixed method exploratory study

**DOI:** 10.3389/fneur.2024.1429928

**Published:** 2024-08-29

**Authors:** Josh W. Faulkner, Elise Callagher, Deborah Snell, Kristopher Nielsen, Molly Cairncross, Alice Theadom

**Affiliations:** ^1^Te Herenga Waka—Victoria University of Wellington, Wellington, New Zealand; ^2^Department of Orthopaedic Surgery and Musculoskeletal Medicine, University of Otago, Christchurch, Christchurch, New Zealand; ^3^Department of Psychology, Simon Fraser University, Burnaby, BC, Canada; ^4^TBI Network, Auckland University of Technology, Auckland, New Zealand

**Keywords:** mild traumatic brain injury, concussion, education, biopsychosocial, post-concussion symptoms

## Abstract

**Introduction:**

Education is strongly advocated as a key component of treatment for mild traumatic brain injury (mTBI) in clinical guidelines. However, there is mixed evidence on the benefit of education. This study aimed to evaluate a new education resource for mTBI. CLARITY is a freely available animated video based on a biopsychosocial conceptualization of mTBI, explaining the complex psychological, environmental and biological mechanisms behind symptoms and recovery.^1^

**Methods:**

24 adults with a history of mTBI participated in this mixed method study to examine prior experience of mTBI education and to evaluate CLARITY. Following viewing of the education video participants’ were invited to engage in a semi-structured interview and to share their perceptions of it via an online anonymous questionnaire.

**Results:**

Thematic analysis of semi-structured interviews revealed one overarching theme: *education is the foundation of recovery*. Participants emphasised the critical role of coherent education in facilitating understanding, engagement in rehabilitation, and positive expectations during recovery. However, the first subtheme was that *existing foundations are weak*. Participants’ previous education was often limited in scope, inconsistent, and delivered in inaccessible ways. The second subtheme was that *new foundations are stronger*. Participants responded positively to CLARITY, highlighting its explanatory biopsychosocial approach, focus on mental health factors and accessible delivery methods as key strengths. Questionnaire responses revealed favourable endorsement of CLARITY’s utility, comprehensibility and accessibility.

**Discussion:**

Recommendations for minor refinements to CLARITY were provided and made, as well as for its use in health care services.

## Introduction

Mild traumatic brain injury (mTBI) occurs after a sudden impact to the head/neck/body, causing acceleration-deceleration trauma to the brain (1). mTBI represents 90% of all traumatic brain injuries (2). Typically resulting in temporary alterations in neurological functioning—i.e., feeling dazed or confused—TBI’s are considered ‘mild’ if they result in no or only brief loss of consciousness (less than 30 min), and less than 24 h of posttraumatic amnesia (3). According to American Congress of Rehabilitation Medicine (ACRM), the term ‘concussion’ is synonymous with mTBI so long as structural abnormalities are neither suspected nor detected on neuroimaging (4). Following a mTBI, individuals may experience post-concussion symptoms such as headaches, dizziness, light and noise sensitivity, difficulties with concentration and executive dysfunction, irritability, depression and anxiety (5). Some individuals may experience persistent post-concussion symptoms (PPCS), typically defined by symptoms that extend beyond 3 months of injury (6). In the past, PPCS was considered to affect only a small subgroup after mTBI—the so-called ‘miserable minority’ (7, 8). However, evidence now indicates that PPCS is much more common. For instance, in a longitudinal study, nearly half of the participants (47.9%) reported four or more post-concussion symptoms 1-year post-injury (9). The consequences of PPCS can be profound, significantly disrupting an individual’s well-being, functioning, and quality of life for months or even years post-injury (10). Consequently, standards for mTBI treatment have evolved to promote earlier proactive interventions (11).

Practise guidelines for mTBI stipulate the importance of providing individuals and their families with education as part of mTBI intervention (12). Education aims to explain what a mTBI is, common symptoms associated with the injury, favourable expectations for recovery and advice about how to manage specific symptoms (13–15). Effective education can equip an individual to face recovery adaptively and empower them to optimally manage their injury (16). However, evidence for the efficacy of education as an intervention for mTBI is mixed. There is evidence that early education reduces post-concussion symptoms (17–19) and improves psychosocial functioning after mTBI (8, 20). Additionally, some systematic reviews have concluded that amongst psychological treatment approaches to mTBI, early education interventions receive the greatest support (21–23). However, other systematic reviews highlight that once methodological weaknesses are accounted for (e.g., high attrition rates, lack of a control group, and poorly conducted randomisation), there is limited robust evidence for the efficacy of educational interventions for mTBI (24–26). These mixed findings may be explained by limitations within existing education approaches in mTBI. More specifically, mTBI education is dominated by two key limitations: (a) the *content* of these interventions is inadequate and outdated, and (b) *delivery* methods do not meet the needs of individuals recovering from mTBI.

In terms of content, current mTBI education is often focused on acute care needs and management of possible medical risks, whilst PPCS and advice for recovery are rarely mentioned (27–29). For those experiencing PPCS, such education may contradict their experiences leading to heightened anxiety and uncertainty (30). Current mTBI education is often frequently based on outdated advice given significant advances in the field over the last 10 years. For example, there is now consistent evidence that complete bedrest does not support beneficial outcomes and may even prolong recovery. As a result, current clinical guidelines suggest rest only for the first 24–48 h, after which individuals should resume normal daily activities at a pace that does not exacerbate symptoms (12, 31–33). Despite this, Kempe et al. (28) found that a majority (72%) of printed education resources advised individuals to ‘rest until asymptomatic’, whilst Silverberg and Otamendi (31) found that clinicians advised the majority of their patients (83%) to take prolonged rest to support recovery from post-concussion symptoms. Current mTBI advice also appears to be largely descriptive in nature (e.g., providing a list of symptoms and blunt ‘one-size fits all’ recommendations) rather than taking an explanatory approach that may allow people to understand their experiences. Qualitative reports by those recovering from mTBI indicate that having a coherent understanding of symptoms and recovery can provide validation and reduce anxiety, whilst facilitating agency, self-esteem and positive recovery expectations (30). Finally, current mTBI education also seems to fail to sufficiently capture a modern biopsychosocial understanding of post-concussion symptoms, and there is some evidence that doing so may reduce catastrophising and facilitate recovery (34, 35).

In terms of delivery, current mTBI education predominantly takes the form of written information such as discharge pamphlets (27, 36–38) or verbal information from clinicians (17, 18, 39). Whilst print resources offer important benefits, such as allowing users to control their learning rate and revisit a tangible source of information, these also come with significant barriers (40). Difficulties with engagement, comprehension, and adherence are barriers to the effectiveness of written health education, especially for those with cognitive difficulties post-mTBI (41–43), and those with limited health literacy (44). Evaluations of mTBI education resources found that less than half meet an acceptable Flesch readability score (i.e., the approximate threshold to be read by 70% of the population) and are primarily written to university or postgraduate level (38, 45). This is particularly concerning as individuals with lower levels of education are particularly vulnerable to sustaining mTBI and experiencing poorer recovery outcomes (46, 47). In comparison to printed or verbal education, education videos have been shown to support better comprehension, adherence and recall (43, 44, 48, 49). For instance, Stalker and Elander (50) found that chronic pain education delivered in a video led to greater improvement in mental-health-related quality of life compared to controls who received standard printed educational resources. At the same time, many health education videos are highly stimulating, fast-paced and contain scientific jargon. These are likely to prove challenging for individuals experiencing ongoing sensory and cognitive difficulties associated with mTBI (51).

In summary, current approaches to mTBI education interventions appear to be insufficient both in terms of the content they contain, as well as the method in which they are delivered. There is a need for mTBI education interventions that are (a) evidence-based, explanatory, and grounded in a biopsychosocial conceptualisation and (b) are appropriate and accessible for individuals with mTBI. To address these limitations, we have developed the Concussion LeaRning And ImplemenTing and recoverY (CLARITY) tool: an education video based on a biopsychosocial conceptualisation of mTBI. The video explains what a concussion is in a way that does not exclude PPCS and articulates key principles of recovery. It is designed to be accessible for individuals who are symptomatic and have low health literacy. In the present study, we used a mixed method design to first contextualise individuals’ experiences of previous mTBI education, and then to evaluate CLARITY’s feasibility and potential value for use in mTBI intervention. More specifically, we aimed to look at the utility (i.e., applicability to mTBI recovery and functional ease of use), accessibility (i.e., with particular consideration of post-concussion symptoms), and comprehensibility (i.e., understandability of communication) for those who have experienced mTBI.

## Materials and methods

### Participants

Eligibility criteria for participants were aged 18 years or older and self-report of having sustained an mTBI. Participants were excluded if they were not fluent in English or had significant visual impairments that would impact their ability to watch the educational video. We recruited participants from an outpatient concussion rehabilitation clinic in the Wellington region of New Zealand and through community advertising via social media posts and posters in public areas. Prior to participating, written consent was obtained. In addition, verbal consent was obtained prior to commencing the interview. Ethics approval was received from the Victoria University Human Ethics Committee: 0000030287.

### CLARITY education video

**Figure 1:**
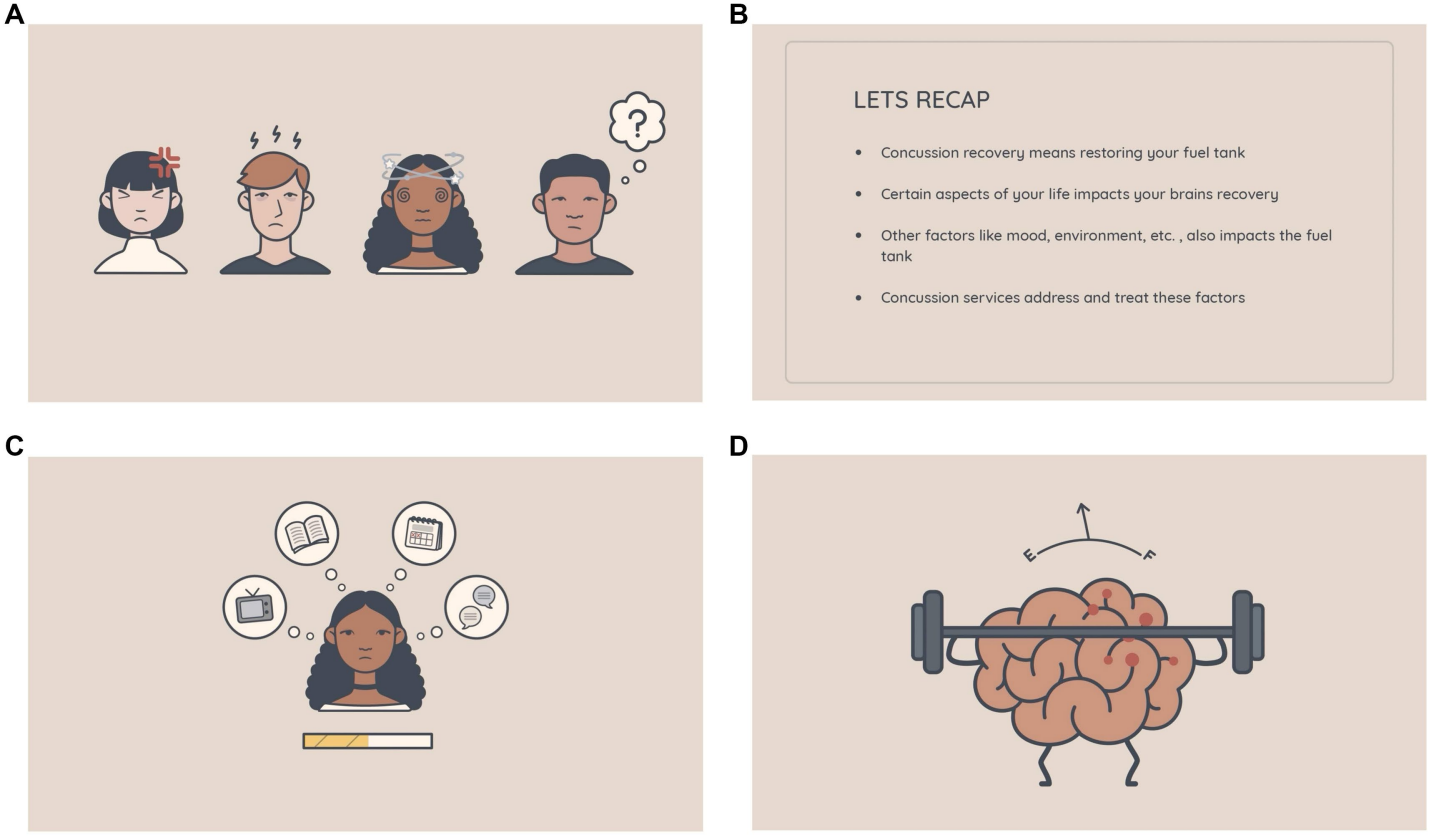
Examples of frames from CLARITY. The video is accompanied by simple animations (as seen in **A**,**C**,**D**) with text displayed only in short summary (‘recap’) sections after the two breaks (as seen in **B**).

Concussion LeaRning And ImplemenTing and recoverY is an animated educational video. It was designed based on current biopsychosocial frameworks of mTBI recovery (52–55), in consultation with international experts in mTBI. CLARITY is 14 min long and, for the purposes of this study, was shown to participants on a computer (but can also be viewed on a phone). It has three parts with a 30-s break between each. The first part contains information regarding what an mTBI is and the temporary changes in an individual’s physiology due to the injury. The second part discusses concussion recovery and provides information on the factors that can contribute to ongoing symptoms. In CLARITY, a specific emphasis is placed on the role mental health has on recovery given robust evidence of the impact psychological factors have on recovery (54, 56, 57). The third and final part provides information on what to expect concerning recovery trajectory and provides some general guidance on symptom management. CLARITY adopts metaphors to aid explanations. For example, the metaphor of a ‘fuel tank’ is used to explain the metabolic changes induced by mTBI and the impact of environmental and psychological stressors on symptoms and recovery. A voice actor provided a slow paced voice-over of the content and the visual content is animated using muted colours with minimal detail and movement. See Figure 1 for examples of the video’s visual delivery.

### Self-report questionnaire

A self-report questionnaire provided a structured anonymous approach to evaluate CLARITY. It consisted of 10 questions on a five-point Likert scale (1 = strongly disagree; 2 = slightly disagree; 3 = neither agree nor disagree; 4 = slightly agree; 5 = strongly agree). Questions were developed in line with previous studies that evaluate education materials utilising insights of their end users (58, 59). The questions assessed three areas: comprehensibility, accessibility and utility. An overview of the questions that pertain to each of these areas is presented in Table 1. The percentage of each response for the questions that comprised each area was calculated.

**Table 1:** Overview of the self-report questionnaire used to evaluate CLARITY.

Area	Questions
*Comprehensibility*	I found the video’s explanations of concussion clear and understandable. I found the video’s explanations of ongoing symptoms after a concussion clear and understandable. I found the video’s explanation of the factors that influence recovery after a concussion clear and understandable.
*Accessibility*	The visual elements of the video helped aide my understanding of ongoing post-concussion symptoms and recovery. I found the video visually appealing overall. The video engaged my attention and interest throughout.
*Utility*	I found the video’s recommendations regarding recovery strategies useful and easily applied. The video was structured in a way that aided my understanding of ongoing post-concussion symptoms and recovery. I would recommend the video to others who have experienced a concussion. I would have found this video helpful in my concussion treatment.

### Procedure

Consenting participants engaged in semi-structured interview guided by an interview schedule (see Supplementary material) either in person or over Zoom. Interview schedules enabled consistency between interviews whilst providing some flexibility to explore areas brought up by the participant not anticipated by the research team (60, 61). In Part 1 of the interview, participants were asked about their previous experiences of education (i.e., the source and extent of education). After the first set of questions in Part 1, participants watched CLARITY displayed on a computer screen. After watching CLARITY, participants were asked to complete a demographic questionnaire and the self-report questionnaire administered via Qualtrics to evaluate CLARITY. In Part 2 of the interview, participants were asked for specific feedback on CLARITY. This included feedback regarding the video’s content, delivery, utility and cultural appropriateness. Participants were also asked to compare CLARITY to their own experiences of concussion and concussion education. Before finishing the interview, participants were prompted to add information they wanted to share that had not already been covered in the interview. Interviews lasted between 25 and 60 min. Audio recordings from interviews were transcribed with Otter.ai (62) automatic transcription software and manually corrected by a research assistant who was external to the research team.

### Qualitative analysis

Inductive thematic analysis was used to analyse participant interviews. This is a qualitative technique wherein researchers work to construct meaning-based patterns (i.e., themes) to construct their interpretation of a qualitative dataset (63). Thematic analysis has previously been used regarding the experiences of individuals with mTBI (30, 64) and by researchers evaluating client experiences of health education tools (59, 65). Given the study’s exploratory nature, an inductive approach was used to categorise themes. With an inductive approach, the analysis is ‘open-coded’, taking a bottom-up approach to best represent meaning as communicated by the participants (60). Unlike a deductive approach, an inductive approach is not intentionally guided by pre-existing theories (66).

As described by Braun and Clarke (60, 66), there are six stages of thematic analysis: (1) familiarisation, (2) generation of initial codes, (3) searching for themes, (4) review of themes, (5) defining chosen themes, and (6) reporting the data. Familiarisation began whilst the transcriptions were checked for errors. After this process was completed, audio recordings were listened to once more, with any initial thoughts about the data noted down. Familiarisation provides the researcher with an entry point into analysis, to facilitate deep engagement with the data and where early analytic ideas are formed (67). Codes were generated and assigned to relevant sections of the data independently by two of the authors (EC and JF) using Microsoft Word’s comment function. After three transcripts were coded, the researchers met to further refine their codes and ensure the process was open and inclusive. The codes and corresponding quotes were imported into Miro to construct initial themes, resulting in an initial thematic map. Both researchers met to examine and combine, cluster or collapse codes into more meaningful patterns. These candidate themes were further refined, clarified and defined.

## Results

### Participants

24 participants engaged in this study and watched CLARITY; 11 participants were seen in-person, and 13 participants were seen over Zoom. A summary of the demographic and injury characteristics is presented in Table 2. The participants ranged in age from 19 to 47, with a mean age of 27.2 (SD = 12.1) years. The majority of participants were female (79.2%) and reported being ethnically New Zealand European (75.0%). Participants were, on average, 47.7 months post-injury. The most common mechanism of injury was a fall (37.6%) and 58.7% of participants had experienced a prior concussion. The majority of participants had received some form of treatment for their mTBI with 61.9% having engaged in an outpatient neurorehabilitation clinic (see Table 2).

**Table 2:** Demographic and injury characteristics of participants (*n* = 24).

Demographic characteristics	
	Mean, Median (SD, 25th and 75th percentile)
**Age**	27.2, 22.5 (12.1, 20, 27)
Gender	*N* (%)
Female	19 (79.2%)
Male	3 (12.5%)
Non-binary	2 (8.3%)
Ethnicity	
NZ European	18 (75.0%)
NZ Māori	2 (8.3%)
Other	4 (16.7%)
Highest education	
Secondary school	8 (33.3%)
Professional qualifications	3 (12.5%)
University	13 (54.2%)
**Injury characteristics**	
	**Mean, Median (SD, 25th and 75th percentile)**
*Time Post Injury (months)*	47.7 (25.6, 32.75, 59.5)
Mechanism of Injury	*N* (%)
Motor vehicle accident	5 (20.8%)
Fall	9 (37.6%)
Hit by object	5 (20.8%)
Other	5 (20.8%)
Treatment	
No	3 (12.5%)
Yes	21 (87.5%)
GP	6 (28.6%)
Hospital/AE	2 (9.5%)
Outpatient Neurorehabilitation Clinic	13 (61.9%)
Previous Concussions	
Yes	14 (58.3%)
No	10 (41.7%)

### Quantitative results

**Figure 2:**
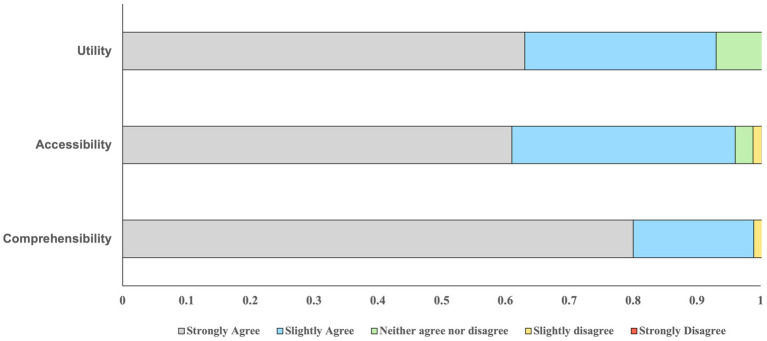
The proportion of responses for each question about CLARITY as it pertains to the areas of evaluation.

As shown in Figure 2, participants’ responses on the self-report questionnaire used to evaluate CLARITY were positive. For the four questions that pertained to utility, 61% of items endorsed by participants were *strongly agree* and 35% of items endorsed were *slightly agree*. 7.3% of items endorsed were *neither agreed or disagreed* and there were no responses indicating disagreement. For the questions that comprised the accessibility and comprehensibility areas, 61 and 80% of items were *strongly agree* respectively, and 35 and 19% of items were *slightly agreed*. Only 1.4% of items within each of these areas were endorsed as *slightly disagree*, and there was no endorsement of *strongly disagree* within these areas.

### Thematic analysis results

Participants’ experiences of previous education and reactions to CLARITY revealed one overarching theme and two subthemes. The overarching theme that *education is the foundation of recovery* reflected how critical education was perceived to be in their recovery. Education formed the basis from which participants saw their injury, sought treatment and engaged with rehabilitation. Sub-theme 1 captured how *Existing Foundations Are Often Weak*, reflecting that the majority of participants’ previous education was insufficient, which had negative implications on their recovery. Sub-theme 2 highlights that the *New Foundations Are Stronger*, as participants reflected that CLARITY is strong both in terms of how the foundations are formed (content) and what material is used (delivery), whilst some tweaks to the foundations could make it even stronger. Each of these themes shall be explored in greater depth in the following sections.

### Overarching theme: education is the foundation of recovery

All participants emphasised that whilst education can have significant positive implications on recovery, when the education is unhelpful or limited it can have negative consequences.

*‘[Education I received from services was] massively helpful. I went from (like) fumbling around in the dark, to (like) actually having a plan to get my life back on track’* (Non-binary, 24 months post-injury).

Participants emphasised that education had a positive effect on how they engaged with rehabilitation and viewed their recovery. For instance, education was crucial in prompting participants to seek further treatment and change their behaviour to support recovery. Education helped them understand and validate their experiences, providing them with a sense of hope that recovery was possible:

*‘[My occupational therapist] would just support me on my understanding of what was happening and basically helped me recover, and (like) gave me confidence that I was going to get over it, and I wasn’t going to be like this forever’* (Female, 47 months post-injury).

On the other hand, limited education had significant negative implications. Many participants reported that a lack of education led to them engaging in behaviours potentially detrimental to recovery:

*‘I just kind of had to figure out my own boundaries. I just kind of have to do a bit of trial and error quite early on. And if you are immediately post-injury (you know) pushing boundaries, maybe too far, it’s maybe (like) detrimental longer term’* (Male, 2 months post-injury).

One participant described that a lack of thorough education led them to engage in risky activities that caused repeated injuries without being aware of the risks involved:

*‘I was told it would take me 6 weeks to heal, and then I’d be done. So I was every time going back and riding a wild horse and I kept getting more [mTBIs]. So I guess there was never a time that we (like) got any information about that and about the problems that would occur if I kept getting repetitive ones’* (Female, 78 months post-injury).

Limited education presented a significant barrier to engaging with further services. For many participants, early education focussing on fixed recovery time-frames and not addressing PPCS meant they were unaware that they could, or should access ongoing support. Some participants also described how a lack of knowledge about the injury, symptoms and recovery led to feelings of uncertainty, frustration, confusion and anxiety:

*‘They told me it was a mild concussion, go home and sleep it off, and because of that, I did not end up getting help until a full year after the first one, having had ongoing problems since’* (Non-binary, 24 months post-injury).

### Sub-theme 1: existing foundations are often weak

The mode of previous education that participants received was mixed. Some participants were given education from medical professionals, whilst others had to source education themselves often from online sources, some even reported receiving no education. Education that was provided was often in either verbal from (i.e., given by a professional) or was a written pamphlet. Of the education that was received, participants were consistently advised to rest and limit their activities. Participants were commonly told to do this for 2–3 weeks and that after this they would be ‘back to normal’,

‘*I was just told that most people feel back to their normal selves after three weeks. And just rest, but do not rest too much. Because then you kind of like, shut down. That’s all I was told by my GP*’ *(Female, 26 months post-injury).*

The education that was provided was also consistently descriptive, rather than explanatory and often took the form of symptom lists and fixed recovery trajectories.

*‘It really is just like a list of things like the best they can do is maybe they are like ‘oh, you know, do this, and then, you know, the next 24–48 h, and then after that, maybe think about these things’. But that’s kind of as far as they go… And it’s not like why or what does this all mean’* (Non-binary, 24 months post-injury).

Participants had mixed reactions about the education they had received. A small number found that the education was helpful, but only when it had come from a specialist (for instance, an occupational therapist). However, the majority of participants found that education was significantly limited, and what they had received was unhelpful. Participants provided several reasons why existing education was unhelpful. This included that the education content did not match their experience, it was generic and vague in content, it often lacked information on what to expect with the recovery, and that it was often provided late in participants’ recovery.

*‘I called [medical healthline] and explained to them kind of all my symptoms. And they were like, ‘Yeah, that sounds like a concussion just like, take it easy’. I was like, ‘Okay’. And that was it. That’s pretty much it. So not really… (Female, 53 months post injury).*

Additionally, participants described the narrow scope of education they received, with some even stating that the sources of education they encountered contained conflicting information. This inconsistency across sources of information spanned from educational information provided by clinicians, others involved in their rehabilitation (e.g., case managers), internet sources, and accounts of mTBI recovery from family, friends, and the media. These inconsistencies contributed to significant uncertainty as participants were unsure of what information to trust. Such experiences were often invalidating of participants’ experiences.

‘[Concussion physician] would always try and say like, ‘Oh, you have (like) post-concussion syndrome’ and then my [family member] and my GP would be like, ‘No, that does not exist (like) that is not a thing’. And so when your medical team is arguing about (like) what you have, and then other people who you trust are also like, ‘Oh, that does not even exist’ (like) what the f*** are they on about?’ (Female, 56 months post-injury).

For a number of participants, the delivery of education presented significant barriers to their ability to engage with and benefit from it. This also included the use of medical terminology that they did not understand. Notably, some participants found that their post-concussion symptoms impacted their ability to read materials, watch online videos or process and remember information.

*‘I was not very good at retaining information immediately after the injury. So they would (like) repeat stuff at subsequent visits and I’d ask the questions and go, ‘I do not get it’’* (Female, 47 months post-injury).

### Sub-theme 2: new foundations are stronger

Overwhelmingly, participants responded positively to CLARITY. Participants felt it would have been helpful during their own recovery noting that it was ‘better’ than the education they had received:

‘*If I had seen that when I got my concussion, it would have been a lot more easy to deal with’* (Female, 76 months post-injury).

Some participants highlighted that the experience of watching CLARITY was valuable even many months after their injury or experiencing symptoms, highlighting that it provided explanation and validation for what they had experienced:

‘*…gave some clarity on what I was actually experiencing, and (like) because I would have like half the people say (like) ‘sure you are’ kind of reaction. So (like) a sense of validation that I’m not, I’m not crazy for feeling like for being sick*’ (Female, 56 months-post-injury).

Participants expressed that a key strength of CLARITY was its explanatory approach and holistic (or biopsychosocial) conceptualisation. Highlighting that it helped them understand the mechanisms of mTBI, fluctuations of symptoms, the importance of returning to activity and factors that support or hinder recovery:

‘*If people were shown this video after hitting their head, instead of just being told to rest and not look at their screens, I think they would have a better understanding of why that is, what that means, what that looks like, kind of thing. And also, a better understanding of like a timeline of (like) this is getting better, I’m good to go back to work or I actually need to get extra help*’ (Non-binary, 24 months post-injury).

Explanations in CLARITY that symptoms are normal, and recovery is possible aided participants to conceptualise recovery from mTBI in an optimistic light:

‘These *are the things that might find difficult’ and it’s normal to be this way… I think it’s nice to know that you can get back to normal*’ (Female, 54 months post-injury).

Many participants found CLARITY’s focus on mental health to be particularly helpful, noting that this was reflected in their own experiences but was not evident in the education they received:

‘I *liked the touching especially on the mental health. Because that was my big thing too, was like depression and anxiety coming out of it and (like) adjusting back to normal life. So I like that that was something because that felt (like) overlooked for the first (like) six months of my recovery*’ (Female, 78 months post-injury).

Participants discussed that the delivery of CLARITY was accessible, and the way it was designed considered the needs of individuals with mTBI. The breaks and recaps were commonly highlighted as beneficial elements of CLARITY, aiding comprehension and attention throughout. Participants appreciated the use of simple terminology and limited visual stimulation (i.e., muted colours and limited movement in animations). All participants noted that the animations and metaphors were particularly effective at both engaging their attention and aiding their understanding of the content.

Participants highlighted that CLARITY could be used by a range of medical professionals, from GPs to concussion services. Additionally, some participants noted that its application would be particularly beneficial when extended to family/caregivers of individuals who had experienced mTBI:

‘*It would be good for partners or (like) people that are with the… it’s kind of more impactful if the partner or the family or friends have seen it, too…Especially if you have (like) gone into an appointment and then suddenly have to go home and you are like, ‘I cannot remember like, what they told me’*’ (Female, 54 months post-injury).

All participants reflected that CLARITY aligned with their cultural background. Specifically, participants noted that the content was accessible and was accompanied by a ‘*mix of representation on the screen*’ (Non-binary, 24 months post-injury). Some highlighted that it would be appropriate for use with adolescents and in low socioeconomic and rural environments due to its accessibility:

‘*I’m just a New Zealand European Pākeha, but I do come from a low socioeconomic background. And I feel like my family who do not have a lot of education, (like) it sounds bad but you know what I mean, (like) I feel like they would understand it completely fine. They would get it*’ (Female, 54 months post-injury).

### Recommendations by participants

Participants noted that CLARITY would be most helpful early in their recovery. However, there were discrepancies as to how ‘early’ was defined. For example, some participants felt it would be most beneficial 1 or 2 days post-injury, whilst others recommended its use up to 2 weeks post-injury. Those who made the latter suggestion highlighted that the severity of post-concussion symptoms may impact an individual’s ability to watch CLARITY, some citing their own difficulties looking at screens for sustained periods. However, participants noted that education in a video format allows individuals to rewatch the resource in their own time and they could modify viewing based on their needs and symptoms (e.g., just listening to the audio).

The majority of critical feedback concerned the video’s length and the amount of content presented. CLARITY’s length was emphasised as potentially problematic as participants had experienced difficulties with sustained attention and memory. However, participants acknowledged the value of all content presented, and most felt it was all needed. To mitigate this issue, participants provided a range of suggested formats. Some participants thought that breaking the video into two or three parts would be helpful; others thought that having CLARITY as one video would be preferable but add a disclaimer regarding its length, emphasising that it can be watched in stages and make use of the breaks when provided:

‘*I think that probably the easiest way for people to retain as much information about it without, I guess getting a bit overwhelmed with everything would probably be to make (like) three parts, so three videos…just because, as I see it, it is a lot to take in and one go and I know if I watched that I would have been interested in it but I just, I would not have been able to watch the whole thing*’ (Non-binary, 34 months post-injury).

Several participants also recommended supplementing CLARITY with an education resource designed specifically for family members about what to expect and how to best support someone after an mTBI. This was highlighted as particularly beneficial for individuals in collectivist cultural contexts:

‘*I remember feeling quite misunderstood and depressed because I do not think [my partner] really understood as well what it was like to have a concussion. It had never happened to him, he’d never experienced it, he’d never been around anyone that had experienced it. So, I think also the educating family members about what your injured family member is going through would be really helpful and how to support them and what to expect as well*’ (Female, 47 months post-injury).

Other minor recommendations included having a written resource alongside CLARITY; displaying keywords throughout the video to reinforce key points; more explanation of what symptoms are (e.g., fatigue); and adding a hypothetical case study. The adoption of participants’ suggestions within CLARITY’s final development shall be explored below (see Recommendations and Clinical Applications).

## Discussion

In this mixed method study, we aimed to (a) understand participants’ experiences of previous mTBI education and (b) evaluate our new education resource: CLARITY. Specifically, we sought to evaluate the utility, accessibility and comprehensibility of CLARITY to appraise its value for use as a mTBI education intervention tool. We did this using a self-report questionnaire and semi structured interviews. Thematic analysis of participants’ interviews revealed the overarching theme that *education is the foundation of recovery*, emphasising that education forms the basis for individuals to understand their injury, guides their recovery, and supports access to appropriate treatment. This theme is consistent with a wealth of evidence highlighting the importance of effective health education (68). When education was comprehensive, aligned with their experience and was delivered by a health professional, participants emphasised it had significant positive implications for their recovery. For these participants, education was vital in prompting further engagement with services, guiding recovery-supporting behaviours, providing hope, validating their experiences, and enhancing their understanding of mTBI symptoms and recovery. Similarly, Snell et al. (30) found an overarching theme that a ‘coherent understanding’ of mTBI symptoms is a driver of positive outcomes, leading to reduced anxiety and facilitating self-esteem, agency, and positive expectations for recovery. Education may be a vessel for a number of biopsychosocial resilience factors that support recovery, such as positive expectations and a sense of agency (69–71).

However, not all foundations provide the same degree of support. Our first sub-theme *existing foundations are often weak,* emphasised that participants’ previous educational experiences were largely insufficient and limited. Participants provided a range of reasons for this. This included the content of the education, as well as the mode in which it was delivered. Education content was often described as vague, generic, descriptive in nature, and lacked explanation on PPCS. Participants also reported that the nature of their post-concussion symptoms impacted their ability to comprehend the education that was often provided in written pamphlets or verbally by a health professional.

Participants also described the negative implications that ineffective education had on their recovery and overall wellbeing. Feelings of anxiety, uncertainty, confusion and distrust were described by our participants. Importantly, these psychological factors can contribute to poorer outcomes and symptom persistence (52, 54, 56, 69). Without a coherent understanding of their symptoms and recovery tools, some participants reported unknowingly engaging in behaviours that were not supportive of recovery, including activities that resulted in repeated injuries. In addition, they often did not access further services as they were unaware they should, or could, do so. The educational experiences described by our participants could help understand why there is mixed evidence pertaining to the effectiveness of mTBI education (24–26, 29).

Our evaluation of CLARITY provides preliminary evidence that this education tool could address limitations in existing mTBI education. More specifically, thematic analysis of participants’ responses after watching CLARITY found that the *new foundations are stronger* both in terms of how the foundations are formed (content) and what material is used (delivery). In support of this, participants provided a positive endorsement of the comprehensibility, accessibility and utility of CLARITY in a self-report questionnaire. Participants emphasised that a key attribute of CLARITY was its explanatory, biopsychosocial conceptualisation of mTBI. They highlighted that this helped them understand mTBI, including: why fluctuations of symptoms occur, the importance of returning to activity, and the breadth of factors that support or hinder recovery. Participants found that CLARITY’s emphasis on the role of mental health factors in recovery was particularly valuable, as it had been largely unexplored by previous education but was a salient feature of their experience. They reflected that having an understanding of these factors would facilitate individual’s agency over their recovery.

These benefits mirror previous findings that suggest that a coherent understanding of biopsychosocial factors in an individual’s injury supports their ability to address these factors. For instance, Morias et al. (35) found that, compared to standard care, a biopsychosocial psychoeducational intervention reduced pain catastrophising, which was further moderated by the adoption of biopsychosocial pain beliefs (over biomedical beliefs). Furthermore, participants stressed that CLARITY’s delivery was clearly designed with consideration of the needs of individuals with mTBI. The breaks and recaps were most commonly highlighted as beneficial elements of CLARITY, aiding comprehension and attention throughout. However, there were some discrepancies in participants’ reflections on the feasibility of the video’s length despite the inclusion of breaks. Participants appreciated the use of simple terminology and metaphors, noting that the content would be understandable for those with low health literacy and/or from a low socioeconomic background. This is a particular strength of CLARITY as a key barrier for clinicians when communicating biopsychosocial conceptualisations of mTBI is their complexity (52).

Despite CLARITY being generally well-received, participants also provided valuable critiques and recommendations for CLARITY’s content, and delivery. First, in regards to content, some participants highlighted that the length and amount of information presented in CLARITY may be a barrier for individuals with cognitive and sensory symptoms of mTBI. At the same time, participants felt that all content was valuable and should not be withdrawn. In response to these concerns, we followed participants’ guidance to add a disclaimer at the beginning of CLARITY advising viewers to pause the video and make use of the breaks provided, and/or listen to the audio alone if required. This resulted in a finalised version of CLARITY, which can be accessed here (see text footnote 1).

Second, participants emphasised that the timing of delivery would be important to the utility of CLARITY. For instance, some participants reflected that if they had received this education early in their recovery, they would have experienced less uncertainty and would not have had to take a ‘trial and error’ approach to managing their recovery. This is in line with findings that suggest that early education shows the most promise in maximising recovery outcomes (21, 25). However, some participants also shared a concern that their cognitive (e.g., memory and attentional processing deficits) and sensory (e.g., light and noise sensitivities) symptoms would have posed barriers to the accessibility of CLARITY early in their recovery. Clinical guidelines advise that individuals abstain from electronic screens in the first 48 h after an mTBI (72). Balancing these concerns, we suggest that individuals first watching of CLARITY would be best at approximately between 7 and 14 days post-injury.

The findings of the present study should be considered in the context of its limitations. Our sample was predominantly female, well-educated and of New Zealand European ethnicity. This limits our ability to generalise our findings and clinical recommendations across the diverse demographic characteristics evident in those who experience mTBI. It will be important to obtain the perceptions of CLARITY in a more diverse cohort, and in an Aotearoa-New Zealand context, this will require a particular focus on Māori. Māori are disproportionately affected by mTBI (73), face poorer outcomes (74) and are more likely to face delayed and inadequate health interventions compared to non-Indigenous communities (74). Māori may benefit from education that employs culturally embedded conceptualisations of mTBI. For instance, a traditional Māori view of mTBI posits the head is the most tapu (sacred) part of the body and that one’s wairua (energy or spirituality) is damaged in tandem to a brain injury (75, 76). Additionally, Elder (77) highlights that family must be considered as the unit that both provides healing and requires healing after a brain injury. Therefore, Māori may have a greater need for extended family to be included in educational interventions for mTBI. Further research should evaluate the education needs of Māori to inform the development of a similarly holistic education to that is culturally and spiritually responsive to Māori individual’s with mTBI and their extended family. Further, participants’ injuries were primarily historical (with a mean of 48 months or 4 years post-injury). Therefore, participants’ reflections may have been subject to recall biases and inaccuracies. For instance, participants may have inaccurately recalled whether watching CLARITY would have posed difficulties in the early stages after their injury. However, despite this, participants were largely able to speak in great detail about their injury, recovery and previous mTBI education, regardless of the time since injury.

Overall, these findings demonstrate that *education is the foundation of recovery* from mTBI, however that content and mode of delivery influences impact. When education is well conceptualised and delivered, individuals are supported by a coherent, useful, and optimistic understanding of their recovery trajectory. In this study, participants’ experiences of existing education was limited in scope, could be contradictory across sources and often inaccessible to those with significant post-concussion symptoms. Based on participant reflections, our biopsychosocial education tool, CLARITY, was found to help provide an explanatory biopsychosocial account of mTBI in an accessible way. This study provides preliminary evidence of the utility, comprehensibility, and accessibility of CLARITY from the perspectives of those who have experienced mTBI. Participants’ feedback informed recommendations for adjustments to the content of CLARITY, as well as its timing and use in healthcare services. The next steps will be to evaluate CLARITY within these services and those who have more recently experienced mTBI.

## Data Availability

The raw data supporting the conclusions of this article will be made available by the authors, without undue reservation.
