# Supplementary material for: Evaluation of a biopsychosocial education resource for mild traumatic brain injury: a mixed method exploratory study
Source: Front Neurol. 2024 Aug 29;15:1429928. doi: 10.3389/fneur.2024.1429928 (PMC11390456; doi:10.3389/fneur.2024.1429928)
Supplement: Supplementary file 1 [file Table_1.DOCX]

| **Supplementary Materials**  Table 1. Semi structured interview schedule. | | | |
| --- | --- | --- | --- |
|  | **Question:** |  | **Potential prompts and follow-up questions:** |
| *Part 1:* | After your concussion, did you receive any education or information about your concussion? |  | If “Yes”: Where did that education come from? (e.g. doctor, specialist, online materials, pamphlet.) How helpful did you find this education/information?  If “No”: Clarify contact with services post-injury, and the extent of information provided. |
|  | Following your concussion experience, what information do you think you would have wanted in your recovery? |  | May need to clarify that this could regard the injury, recovery or anything else related to their experiences. |
|  | ***Participants watched CLARITY*** | | |
| *Part 2:* | Overall, how did you find the concussion video? |  | Bridge to the next question: which of the things that you have mentioned... |
|  | Was there anything in the video that you found particularly helpful or informative? |  | How did that aid your understanding of [concept]?  Try to discern what about the information or delivery facilitated their understanding? |
|  | Was there anything in the video that you found confusing or hard to understand? |  | Try to discern what about the information or delivery was a barrier to their understanding? [language; analogies; missing information] |
|  | What did you like the most about the ways the video was delivered? |  | Prompt for specifics about the form of delivery [visual elements, video format, language used]. |
|  | If you could change anything about the video, what would it be? |  | May need to clarify reasons behind changes.  What solutions/alternatives would make it easier to understand? |
|  | Did you find that the explanations and examples used in the video were appropriate to your cultural background? |  | Explore what and why something may be culturally inappropriate.  Prompt for potential solutions/alternatives. |
|  | Based upon your own experience of concussion, was there any important information missing that you expected to see? |  | Do you have an understanding of [concept] that is different to how it was explained in the videos? |
|  | When do you think that watching this video would have been most helpful, after your concussion? |  | Clarify reasoning behind timing suggestion. |
|  | If you received concussion treatment, how does this education compare to what education you may have received? |  | Was there any information/recommendations that you had not previously encountered?  Prompt for education about mood and mental health factors? |
|  | Is there anything else you would like to add about your experience watching the educational video? |  | Probe for any further recommendations. Use as opportunity to prompt if they have further questions. |
